# Post-hospitalization course and predictive signs of suicidal behavior of suicidal patients admitted to a psychiatric hospital: a 2-year prospective follow-up study

**DOI:** 10.1186/1471-244X-12-186

**Published:** 2012-10-31

**Authors:** Naoki Hayashi, Miyabi Igarashi, Atsushi Imai, Yuka Yoshizawa, Kaori Utsumi, Yoichi Ishikawa, Taro Tokunaga, Kayo Ishimoto, Hirohiko Harima, Yoshitaka Tatebayashi, Naoki Kumagai, Makoto Nozu, Hidetoki Ishii, Yuji Okazaki

**Affiliations:** 1 Department of Psychiatry, Tokyo Metropolitan Matsuzawa Hospital, Tokyo, Japan; 2 Schizophrenia Research Team, Tokyo Metropolitan Institute of Medical Science, Tokyo, Japan; 3 Faculty of Medicine, Tokyo Medical and Dental University, Tokyo, Japan; 4 Department of Neuropsychopharmacology, National Institute of Mental Health, National Center of Neurology and Psychiatry, Tokyo, Japan; 5 Osaka Prefectural Tondabayashi Health Center, Osaka, Japan; 6 Affective Disorders Research Team, Tokyo Metropolitan Institute of Medical Science, Tokyo, Japan; 7 Disabled Persons Programs Division, Bureau of Social Welfare and Public Health, Tokyo Metropolitan Government, Tokyo, Japan; 8 Tokyo Metropolitan Tama Comprehensive Center for Mental Health and Welfare, Tokyo, Japan; 9 Graduate School of Education and Human Development, Nagoya University, Nagoya, Japan; 10 Michinoo Hospital, Nagasaki, Japan

**Keywords:** Suicide attempt, Self-injurious behavior, Follow-up studies, Psychiatric hospitals, Mental disorders

## Abstract

**Background:**

Suicidal patients admitted to a psychiatric hospital are considered to be at risk of suicidal behavior (SB) and suicide. The present study aimed to seek predictors of SB recurrence of the high-risk patients by examining their post-hospitalization course.

**Method:**

The design was 2-year prospective follow-up study of patients consecutively admitted with SB to a psychiatric center in Tokyo. The DSM-IV diagnoses and SB-related features of subjects were determined in structured interviews. Subsequently, the subjects underwent a series of follow-up assessments at 6-month intervals. The assessment included inquiries into SB recurrence, its accompanying suicidal intent (SI) and SF-8 health survey. Analyses of serial change over time in the follow-up data and Cox proportional hazards regression analyses of SB recurrence were performed.

**Results:**

106 patients participated in this study. The dropout rate during the follow-up was 9%. Within 2 years, incidences of SB as a whole, SB with certain SI (suicide attempt) and suicide were 67% (95% CI 58 - 75%), 38% (95% CI 29 - 47%) and 6% (95% CI 3 - 12%), respectively. Younger age, number of lifetime SBs and maltreatment in the developmental period were predictive of SB as a whole, and younger age and hopelessness prior to index admission were predictive of suicide attempt. Regarding diagnostic variables, anxiety disorders and personality disorders appeared to have predictive value for SB. Additionally, poor physical health assessed during the follow-up was indicated as a possible short-term predictor of SB recurrence.

**Conclusions:**

This study demonstrated a high incidence of SB and suicide and possible predictors of SB recurrence in the post-hospitalization period of psychiatric suicidal patients. Specialized interventions should be developed to reduce the suicide risk of this patient population.

## Background

Suicidal behavior (SB) is a major health problem worldwide. It is usually considered to emerge from a burden of mental illness, and therefore, managing SB is an important responsibility for mental health workers. SB has been an established predictor for completed suicide [1-5] whereas other predictive factors, such as psychiatric disorders, especially mood disorders and schizophrenia [3,6,7], and a history of psychiatric hospitalization [8], are also of clinical importance. A nationwide psychological autopsy study [2] demonstrated that a high rate, 44%, of suicide victims had a history of previous SB. A review on follow-up studies [9] indicated that approx. 7% of patients who presented with SB in an emergency department died of suicide within the following 9 years. Salient elevation of suicide risk by a history of SB was recognized in a large-scale patient register-based study [3]. Some features of SB such as intensity of accompanying suicidal intent (SI) [10,11] and SB repetition [12] have also been indicated to have value for suicide prediction. Consequently, SB evidently comprises a critical part of the process that lead to suicide completion, which is known as “a pathway to suicide” [13] , “suicidal process” [14] and acquisition process of “the capability for suicide [15]” in many cases.

Considering the importance of SB, a great number of studies have investigated predictors of SB in a lifetime history and clinical course of psychiatric patients by using various methodologies, and found a wide range of signs or factors antecedent to SB. A history of SB or SB repetition has been a commonly found predictive sign of subsequent SB [16-23]. Psychiatric disorders and symptoms including severity of depression [16,23], psychotic disorders [24,25], personality disorders (PD) [26,27], substance abuse [28], and hopelessness [16,20,27,29] were recognized as predictors in previous studies. Socio-demographic factors such as female gender, younger age [23,27] and unemployment [18,19,25], and factors in lifetime history such as childhood maltreatment [22,25,28,30,31], have been highlighted in investigations. Protective factors such as social support and living with a partner [23] are also considered to have an influence on SB occurrence. If these signs and factors could be understood in terms of their positions in the processes leading to suicide [13-15], it would greatly contribute to the development of suicide prevention strategies for the patients. However, the number of studies that provide information for that purpose still remains insufficient since they need to cover the processes of various patient populations and phases of their clinical course.

The present study aimed to investigate the post-hospitalization course of psychiatric suicidal patients while putting the focus on recurrence of SB. The studied sample was characterized by a predominance of involuntarily admitted patients and the majority diagnoses of mood disorders, anxiety disorders and PDs as shown in the cross-sectional study [32] that we had conducted as the former stage of the investigation on hospitalized suicidal patients, most of whom subsequently participated in this study. In the follow-up stage, a series of assessments were performed during the 2-year study period at 6-month intervals.

This study addresses the following questions about the discharged suicidal patients: what characteristics are predictive of SB recurrence, what are essential in SB prediction among the characteristics, and whether there are short-term (3-month) predictors of SB other than the predictors of within 2-year SB recurrence. In the analyses, suicide attempt (SB accompanied with suicide intent (SI)) was included in target variables since the presence or absence of SI would be of importance in classifying SB types [33,34]. In addition, clinical characteristics of subjects who died of suicide within the 2-year period were examined. It is expected that predictive characteristics of SB that this study attempts to identify in the clinical course be usable for contriving SB and suicide prevention programs for this high-risk population.

## Methods

### Subjects

The patients included in the study were those who were consecutively admitted to Tokyo Metropolitan Matsuzawa Hospital (TMMH) during a 20-month period from April 2006 to November 2007 with SB during the 2 weeks prior to admission, and were subsequently discharged to a residence within a district in Tokyo, for which mental health administration Tokyo Metropolitan Chubu Center for Mental Health and Welfare (TMCMW) is responsible. TMMH and TMCMW are located on the same campus, and comprise a psychiatric center for psychiatric emergency and other regional services within a mixed business and residential area in Central Tokyo.

In identifying SB, we applied at the start of this investigation the definition of “non-fatal suicidal behavior, with or without injuries” by de Leo et al. [35], “A non-habitual act with non-fatal outcome that the individual, expecting to, or taking the risk, to die or to inflict bodily harm, initiated and carried out with the purpose of bringing about wanted changes.” This concept might cause a problem in the investigation since it included various related concepts such as suicidal attempt and self-harm without SI [33,34] or non-suicidal self-injurious behavior [36]. To minimize the problem, the level of SI that accompanied SB was consistently to be taken into account in this study.

The inclusion criteria of the subjects were (1) age at index admission equal to 20 years or more, (2) a hospital stay longer than 3 days, (3) absence of prominent mental retardation or organic brain damage, (4) fluent in Japanese, (5) able to comprehend the study procedures and to undergo entry assessments safely, (6) discharged to a residence in an area within the reach of a follow-up contact and assessment, and (7) provided written informed consent for study participation, and in cases of involuntary admission, additional consent from the patient’s family or guardians.

### Assessment

The assessments included in the entry interviews were as follows.

(1) *Suicidal behaviors (SBs) prior to index admission and in lifetime history*: Methods of SB used immediately prior to admission, and methods, numbers and time points of SBs in lifetime history were recorded. The 16 SB methods classified on the basis of the report of the 2004–2006 Japanese Ministry of Health, Labor and Welfare supported research by Hosaka et al. were individually used for inquiry in the interview. Presence or absence of the following 5 most frequent SB methods was used in the analyses: self-cutting, overdosing, self-strangulation, attempting traffic death and jumping from a height. The total number of lifetime SBs before index admission was also counted.

(2) *Structured Clinical Interview for DSM-IV Axis I Disorders, Clinician Version (SCID-I, CV)*[37]*and Structured Clinical Interview for DSM-IV Axis II Personality Disorders (SCID-II)*[38]: Psychiatric and personality disorders of the subjects based on the Diagnostic and Statistical Manual of Mental Disorders, Fourth Edition (DSM-IV), were determined by conducting SCID-I CV and SCID-II. Principally, 7 major DSM-IV diagnostic groups: mood, psychotic, substance-related and anxiety disorders, and clusters A, B and C of PDs, were used in the analysis.

(3) *Suicide Intent Scale (SIS)*[39]: SIS is a 20-item semi-structured instrument designed to record information concerning a suicidal individual’s desire to die by inquiring into circumstances and patient’s reports of thoughts and feelings at the time of SB. In this study, a scale composed of the first 15 SIS items was used to rate the intensity of SI.

(4) *Beck Depression Inventory-II (BDI)*[40]*and Beck Hopelessness Scale (BHS)*[41]: BDI is a widely used 4-point, 21-item self-report scale developed for assessing depressive symptoms. BHS, a self-report scale for use in measuring hopelessness, is composed of 20 true-false items. Both scales were verbally administered in the entry interviews to assess the levels of depressive symptomatology and hopelessness in the subjects during 2 weeks prior to index admission.

(5) *History of maltreatment before the age of 18 years*: To assess the history of maltreatment during development, a 3-point (absent, unclear, and certainly present), 7-item semi-structured interview was devised for use in this study. The types were intra- and extra-familial sexual maltreatment, intra- and extra-familial physical maltreatment, intra- and extra-familial verbal maltreatment and intra-familial neglect before the age of 18 years. With an exception of sexual maltreatment, all types had to be present for a duration of 1 month or longer to be considered “present”. Only “certainly present” was counted as a positive response for each of the items. Presence or absence of a positive response to any of the maltreatment items was used in the analyses.

Additionally, socio-demographic data of the subjects were recorded. Dichotomous variables of unemployment, marital status (never married and living with a partner), education (high-school graduate or higher) and living alone prior to index admission were used in the analyses.

The entry assessment was performed over more than one interview to avoid much exhaustion of the subjects. The 10 interviewers were TMMH psychiatrists and psychiatric residents with more than 2 years of clinical experience in psychiatry. They received more than 10 training sessions before conducting the assessments. All of the assessments were individually reviewed by our study group. Details regarding a cross-sectional investigation of admitted suicidal patients, including the subjects in this study, are provided elsewhere [32].

The assessments in the follow-up interviews conducted at 6-month intervals were as follows.

(1) *SB recurrence*: Methods, frequencies and time points of SBs, their accompanying SI, and physical treatment for damage caused by SB within a 6-month period prior to the interview were recorded. SI was inquired about using a 3-point (absent, unclear or minimal, and certainly present) single scale. SB that was accompanied with “certainly present” SI was defined as SB with SI (suicide attempt) in this study. The numbers and time points of SB or SB with SI observed during the follow-up period were used in the analyses.

(2) *Service utilization and social support*: Utilization of psychiatric and medical services, including hospitalization, outpatient treatment, medication, and rehabilitation activities, was inquired about. Presence or absence of family members, relatives, and other persons on whom the subject could rely for help, as well as their satisfaction with the help and support obtained, was assessed. In the analyses, regular attendance for outpatient treatment (more than once a month) and presence or absence of individuals on whom the subject could rely for help were used, which were supposed to represent quality of treatment and social support, respectively.

(3) *SF-8 health survey, standard version*[42]: SF-8 health survey is a well-validated instrument of health-related quality of life. It provides a concise assessment of general physical and mental conditions, which comprises 8 inquiries about physical and mental health conditions and distressing symptoms such as pain, anxiety and depression during the preceding 1-month period. The items are scored on a 5- or 6-point Likert scale ranging from very poor to excellent. Standardized SF-8 physical and mental component summary scores (PCS and MCS scores) were calculated by using the scoring algorithm provided for the instrument [43]. Higher scores on the SF-8 PCS and MCS scales indicated subject’s better physical and mental health, respectively.

(4) *BHS, 4-item version*[44]: BHS, 4-item version, was used for the follow-up assessments. This version has been proposed as a possible substitute for the original BHS with comparable validity by Aish and Wasserman [44]. Higher scores of this scale indicated greater hopelessness.

The day of completing the entry assessment was set as the start of follow-up. Clinical psychologists and public health nurses of TMCMW and psychiatrists of TMMH conducted the follow-up assessments principally by interviewing the subjects. If subjects were unable to attend the interview in person, inquiries and assessments were made through mail or telephone surveys. In addition, information from family members who provided the consent for study participation at entry was used.

Since the subjects were considered to be at risk of suicide, the following supports were provided for them. During the follow-up period, a telephone consultation help desk was set up in TMCMW. In the assessment, when necessary, the interviewers were permitted to introduce available treatment and social support resources to the subjects.

### Statistical analysis

The statistical analysis for this study included several stages. First, serial changes during the follow-up period in clinical variables: numbers of SB as a whole and SB with SI, SF-8 PCS and MCS scores and BHS 4-item version score, were examined by applying repeated measures ANOVA and Friedman test for normally and non-normally distributed data, respectively. Second, univariate Cox proportional hazards regression analyses of SB as a whole and SB with SI during the follow-up period were performed to investigate the association with demographic, life-historical, clinical and diagnostic variables at the entry assessment. Subsequent multivariate analyses with a forward conditional stepwise procedure (Entry: p<0.05, Removal: p<0.10) were conducted by using 2 data sets of the diagnostic group variables and all the variables that were found to be significant in the previous univariate analyses. Third, for the purpose of investigating clinical variables associated with short-term SB recurrence, stepwise Cox proportional regression analyses of SB as a whole and SB with SI within a subsequent 3-month period were conducted with the variables of data obtained in the follow-up assessments: SF-8 PCS and MCS scores, BHS 4-item version scores, regularity of outpatient clinic attendance, and presence of individuals on whom the subjects could rely for help as possible covariates. For this portion of the analyses, the variables that were significantly associated with SB recurrence in the previous multivariate analyses using all the variables were also included as covariates to control their effects on short-term prediction. In the analyses, completed suicide was included in SB with SI.

In statistical analyses, a significance level of 0.05 was applied. The statistical package SPSS Release 16.0.2 (SPSS Inc., Chicago, IL, USA; 2008) was used for the entire analyses. This study was approved by the ethical committee of TMMH on 28 Mar, 2006.

## Results

### Clinical characteristics of the subjects at entry

Of a total of 3450 admissions to TMMH during the 20-month study period, 292 cases (280 patients) with SB were identified. 176 patients fulfilled criteria (1)-(6) for participation in this study. The numbers of patients excluded from the studied sample according to the criteria items were 18, 24, 8, 9, 33 and 12 in the order of criteria (1)-(6). In 59 cases, the patient or the family guardian did not give consent for study participation (criterion (7)), 2 did not complete the entry assessment, and 9 did not respond to any request for follow-up contact. As a result, 106 patients (60% of the patients eligible for inclusion) participated in at least one of the follow-up assessments. Averages (SDs) of the hospital stay of index admission and duration of the period between admission and end of entry assessment for the subjects were 42.3 (26.3) days and 20.6 (13.6) days, respectively. The admission type of 81 subjects (76%) was involuntary.

A comparison of the 106 subjects included in this study with the remaining 70 eligible but not participating patients, with respect to demographic data, treatment history, diagnoses based on International Classification of Diseases 10^th^ version (ICD-10) in the record of TMMH and the 5 most frequent SB methods, revealed a significant difference in the percentage of psychotic disorders (ICD-10 code: F2) (33% vs. 49%, Chi square=4.28, df=1, p=0.039). This finding suggests that the subjects in this study had a lower rate of psychotic disorders.

The 106 participants in this study were composed of 48 males and 58 females. The mean age (SD) was 36.6 (11.6) years at the entry assessment. Marital states were never married 59 (55%), living with a partner 28 (26%) and widowed, separated or divorced 20 (19%). Education levels were less than high school graduate 27 (26%), high school graduate 47 (44%) and college graduate or higher 32 (30%). 37 subjects (35%) were living alone. Employment conditions were fulltime worker 16 (15%), part-time worker 21 (20%) and unemployed 52 (49%). 91 subjects (85%) had been continuing psychiatric outpatient treatment before index admission. 64 (60%) had a history of psychiatric hospitalization. Overall, high percentages of unemployment, living alone and continuous use of psychiatric services characterized the sample.

SB methods exhibited by the subjects immediately prior to index admission were self-cutting 40 (38%), overdosing 37 (35%), self-strangulation 13 (12%), attempting traffic death 11 (10%) and jumping from a height 10 (9%). Other SB methods numbered only less than 4 (4%). 25, 50 and 75 percentiles (range) of the total number of SBs in lifetime history of the subjects were 4, 7 and 21.25 (1–141), respectively.

Tables 1 and 2 present DSM-IV axis I disorders and axis II PDs of the subjects. Mood disorders, anxiety disorders and borderline PD were presented by more than half of the subjects. 91 (86%) of the subjects were diagnosed with at least one PD.

**Table 1:** DSM-IV Axis I disorders of the subjects at entry (N=106)

	**N**	**%**
Mood disorders	70	66
Major depressive disorders	46	43
Dysthymic disorder	4	4
Bipolar I disorder	7	7
Bipolar II disorder	12	11
Anxiety disorders	66	62
Panic disorders	43	41
Specific phobia	11	10
Social phobia	5	5
Obsessive-compulsive disorder	11	10
Post-traumatic stress disorder	18	17
Generalized anxiety disorder	11	10
Substance-related disorders	40	38
Alcohol use disorders	26	25
Non-alcohol use disorders	21	20
Psychotic disorders	26	25
Schizophrenia	19	18
Schizoaffective disorder	3	3
Brief psychotic disorder	4	4
Eating disorders	12	11

**Table 2:** DSM-IV personality disorders (PDs) of the subjects at entry (N=106)

	**N**	**%**
Cluster A personality disorders (PDs)	35	33
Paranoid PD	18	17
Schizoid PD	17	16
Schizotypal PD	9	9
Cluster B personality disorders (PDs)	72	68
Antisocial PD	28	26
Borderline PD	65	61
Histrionic PD	8	8
Narcissistic PD	13	12
Cluster C personality disorders (PDs)	54	51
Avoidant PD	36	34
Dependent PD	14	13
Obsessive-compulsive PD	25	24

Averages (SDs) of SIS, BDI and BHS scores were 11.5 (5.9), 31.5 (11.9), and 13.6 (5.9), respectively. The results indicated that the subjects exhibited a severe level of SI, depressive symptoms and hopelessness prior to index admission, which were comparable to those of suicidal patients in medical or emergency settings [32]. Particularly, 93 (88%) of the subjects reported the presence of SI component (SIS Item 13). From 13 subjects who denied SI in the entry assessment, SI that accompanied SB immediately prior to index admission was heard by family members or others around them. Therefore, the SBs could be understood as suicidal attempts.

A history of any maltreatment before the age of 18 years was reported by 66 (62%) of the subjects. Those who had experienced sexual, physical and verbal maltreatment, and neglect were 16 (15%), 40 (38%), 57 (54%) and 20 (19%), respectively.

### Post-hospitalization course and incidence of SB and suicide

Data of the whole 2-year clinical course variables and complete data of all the variables were obtained from 96 subjects (including deceased ones) and 65 subjects, respectively. In a total of 341 follow-up contacts, interviews, correspondences through mail and telephone, and hearing from family informants were 233 (69%), 83 (24%), 8 (2%) and 16 (5%), respectively. Readmission to a psychiatric hospital was seen in 65 (61%) subjects within the 2-year period. In 289 (85%) of 339 assessment contacts, regular outpatient treatment attendance (more than once a month) was recorded.

In a total of 526 SBs recorded during the follow-up, SB methods were self-cutting 161 (31%), overdosing 157 (30%), self-strangulation 119 (23%), attempting traffic death 15 (3%) and jumping from a height 31 (6%). Levels of SI that accompanied SB were “certainly present” 258 (49%), “unclear” 190 (36%) and “absent” 78 (15%). 278 SBs (53%) necessitated medical treatment for the physical damage.

6 deaths by suicide were identified during the follow-up. A very high suicide rate per year of 3.1% (95% confidence interval (CI) 1.4 - 6.5%) was calculated from within 196 person-years of observation. Methods of suicide were hanging (1 person), jumping from a height (1 person), traffic death (1 person), overdosing (1 person) and an unknown method (2 persons). The suicide victims were 3 males and 3 females: 3 females in their twenties or thirties with mood and anxiety disorders and cluster B PDs, 2 of whom also had substance-related disorder, 1 male in his thirties with schizophrenia, anxiety disorder and cluster B PD, and 2 males in their sixties with schizophrenia. Additionally, 1 male in his sixties died from physical disease.

**Figure 1:**
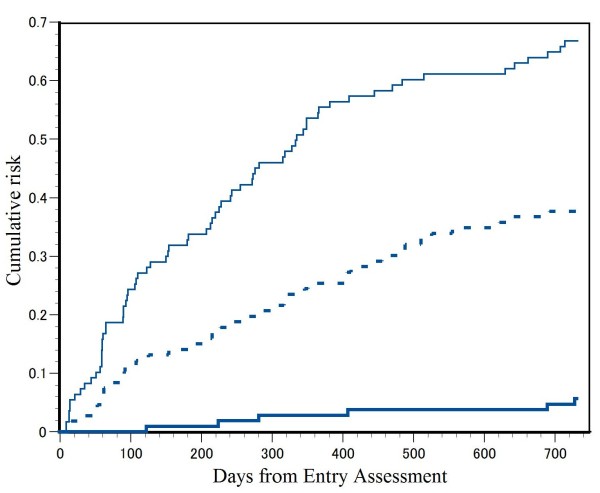
Cumulative risk of suicidal behavior (SB) (thin line) suicide attempt (SB with suicidal intent (SI)) (dashed line) and suicide (thick line).

Figure 1 displays the cumulative incidence rate of SB as a whole, SB with SI and suicide during the follow-up period for the total cohort. Incidence rates of SB, SB with SI and suicide within 1 year were 53% (95% CI 43 - 62%), 27% (95% CI 18 - 35%) and 2.8% (95% CI 1.0 - 8.0%), and those within 2 years were 67% (95% CI 58 - 75%), 38% (95% CI 29 - 47%) and 5.7% (95% CI 2.6 - 11.8%), respectively. The median for days between the start of follow-up and the recurrence of SB as a whole was 334 days (95% CI: 252–416).

Table 3 presents the serial changes in averages of the numbers of SB as a whole and SB with SI, and the scores of SF-8 PCS, SF-8 MCS and BHS 4-item version recorded in follow-up assessments. Repeated measures ANOVAs of the serial scores found a significant linear component in SF-8 MCS scores and BHS 4-item version scores in addition to their significant time effects. The findings indicated an improvement in distressing mental symptoms and hopelessness during the follow-up period. In contrast, statistical tests for examining SF-8 PCS score and the numbers of SBs and SBs with SI did not yield significant results.

**Table 3:** Suicidal behavior recurrence and measures of physical and mental conditions in the follow-up assessments

	**1**^**st**^**assessment**	**2**^**nd**^**assessment**	**3**^**rd**^**assessment**	**4**^**th**^**assessment**
Number of SB as a whole ^a^	1.40	1.16	1.12	1.38
Number of SB with SI ^a^	0.64	0.73	0.61	0.46
SF-8 PCS score (SD) ^b^	47.9 (9.4)	48.7 (8.8)	47.6 (9.0)	48.1 (7.9)
SF-8 MCS score (SD) ^b, c^	37.0 (10.8)	40.3 (12.1)	41.2 (9.7)	42.6 (9.5)
BHS 4-item version score (SD) ^b,c^	2.61 (1.26)	2.30 (1.42)	2.28 (1.34)	2.08 (1.42)

SB: suicidal behavior, SI: suicide intent, Number of SB (with SI): number of SB (with SI) during the previous 6 month period, SF-8 PCS score: SF-8 physical component summary score, SF-8 MCS score: SF-8 mental component summary score, BHS: Beck Hopelessness Scale.

^a^ Means of 89 not-deceased subjects with the whole 2-year clinical course data set.

^b^ Means of 65 subjects with the complete data set.

^c^ Repeated measures ANOVAs found a significant time effect and linear component in SF-8 MCS scores (Wilks’ lambda=0.798. F_3, 62_=5.22, p=0.003 and F_1, 64_=14.83, p<0.001, respectively) and BHS 4-item version scores (Wilks’s lambda=0.877, F_3, 62_=3.12, p=0.42 and F_1, 64_=7.00, p=0.010, respectively).

### Association of subsequent BSs with clinical variables

Table 4 presents the results of univariate Cox proportional hazards regression analyses examining the recurrence of SB as a whole and SB with SI, with demographic and clinical variables as a covariate. SB as a whole was associated with age and unemployment at entry, number of lifetime SBs, and maltreatment before the age of 18 years, anxiety disorders and cluster B PD. SB with SI was associated with age at entry, and scores of BDI and BHS. However, none of 5 SB methods, marital status, education and living alone on index admission was associated with SB during the follow-up.

**Table 4:** Univariate Cox proportional hazards regression analyses of suicidal behavior (SB) recurrence during the 2-year follow-up period

**Variable**	**Suicidal behavior (SB)**			**SB with suicide intent (SI)**		
	**Exp(B)**	**95% CI**	**p**	**Exp(B)**	**95% CI**	**p**
Age at entry	0.958	0.935 - 0.981	<0.001	0.962	0.936 - 0.998	0.028
Female gender	1.007	0.631 - 1.607		0.816	0.439 - 1.520	
Living with a partner	0.654	0.369 - 1.157		0.971	0.485 - 1.945	
Unemployment	1.766	1.103 - 2.828	0.018	1.820	0.966 - 3.427	0.060
Number of lifetime SBs	1.016	1.010 - 1.023	<0.001	1.007	0.999 - 1.016	0.079
Maltreatment ^a^	2.611	1.535 - 4.439	<0.001	1.536	0.781 – 3.021	
SIS score	1.001	0.962 - 1.042		1.042	0.990 - 1.097	
BDI score	1.014	0.994 - 1.035		1.036	1.006 - 1.067	0.014
BHS score	1.042	0.987 - 1.101		1.123	1.034 - 1.220	0.006
Mood disorders	1.207	0.738 - 1.975		1.265	0.643 - 2.488	
Psychotic disorders	0.681	0.390 - 1.190		0.888	0.423 - 1.865	
Substance-related disorder	1.359	0.846 - 2.184		1.426	0.764 - 2.661	
Anxiety disorders ^b^	2.165	1.288 - 3.639	0.004	1.948	0.952 - 3.986	0.068
Cluster A PD ^c^	1.286	0.786 - 2.104		1.902	1.015 - 3.564	0.045
Cluster B PD ^d^	2.341	1.338 - 4.098	0.003	1.278	0.638 - 2.558	
Cluster C PD	1.275	0.800 - 2.033		1.129	0.607 - 2.101	

95% CI: 95% confidence interval, Number of lifetime SBs: number of SBs in lifetime history, maltreatment: presence of maltreatment before the age of 18 years, SIS: Suicide Intent Scale, BDI: Beck Depression Inventory, BHS: Beck Hopelessness Scale, PD: personality disorder.

^a^ The maltreatment subcategories that had significant associations with recurrence of SB were physical maltreatment, verbal maltreatment and neglect (Exp (B) 1.860, 95% CI 1.161 - 2.981, p=0.010, Exp (B) 2.390, 95% CI 1.460 - 3.912, p=0.001, and Exp (B) 2.602, 95% CI 1.506 - 4.495, p=0.001, respectively).

^b^ Diagnoses in this diagnostic group variables that showed a significant association with recurrence of SB were panic disorder with agoraphobia, obsessive-compulsive disorder, posttraumatic stress disorder and specific phobia (Exp (B) 2.405, 95% CI 1.269 - 3.492, p=0.004, Exp (B) 2.912, 95% CI 1.511 - 5.612, p=0.001, Exp (B) 2.048, 95% CI 1.153 - 3.637, p=0.014 and Exp (B) 2.038, 95% CI 1.037 - 4.005, p=0.039, respectively).

^c^ PD in this group that showed a significant association with SB with SI was paranoid PD (Exp (B) 2.579, 95% CI 1.287 - 5.169, p=0.008).

^d^ PDs in this group that showed a significant association with SB as a whole were antisocial PD, histrionic PD and borderline PD (Exp (B) 2.017, 95% CI 1.220 - 3.332, p=0.006, Exp (B) 1.735, 95% CI 1.064 - 2.888, p=0.027, and Exp (B) 2.523, 95% CI 1.186 - 5.366, p=0.016, respectively).

Table 5 presents the results of stepwise Cox proportional hazards regression analyses of SB as a whole and SB with SI. The analyses using diagnostic variables as possible covariates indicated that anxiety disorders and cluster B PD had a significant association with SB as a whole. Regarding SB with SI, no significant association with psychiatric disorders were found. Analyses using all the variables with a significant association demonstrated that SBs as a whole was associated with age at entry, maltreatment before the age of 18 years and number of lifetime SB, and that SB with SI was associated with age at entry and hopelessness prior to index admission. Overall, SB as a whole and SB with SI indicated a rather different pattern of associations: SB as a whole was associated with life historical and life-situational variables, and SB with SI, mainly with depressive symptoms prior to index admission.

**Table 5:** Stepwise Cox proportional hazards regression analyses of suicidal behavior recurrence during the 2-year follow-up period

**Variable**	**Suicidal behavior (SB)**			**SB with suicide intent (SI)**		
	**Exp(B)**	**95% CI**	**P**	**Exp(B)**	**95% CI**	**p**
Diagnostic group variables						
Anxiety disorders	2.055	1.122 - 3.765	0.020			
Cluster B PD	1.840	1.053 - 3.214	0.032			
All variables						
Age at entry	0.953	0.927 - 0.980	0.001	0.963	0.930 - 0.997	0.032
Maltreatment	2.655	1.512 - 4.662	0.001			
Number of lifetime SBs	1.011	1.004 - 1.018	0.002			
BHS score				1.120	1.037 - 1.222	0.005

95% CI: 95% confidence interval of Exp (B), PD: personality disorder, Diagnostic group variables: presence or absence of 7 diagnostic group: mood, psychotic, substance-related and anxiety disorders, and clusters A, B and C of PDs, All variables: all the variables that showed a significant association in the previous univariate analyses (see Table 4), Maltreatment: presence of any maltreatment before the age of 18 years, Number of lifetime SBs: Number of SBs in lifetime history, BHS: Beck Hopelessness Scale.

In the Cox proportional hazards regression analyses of SB as a whole and SB with SI within the 3 months subsequent to a follow-up assessment, the variables that indicated a significant association in the previous multivariate analyses of SBs using all the variables with a significant association, were included to control their effects on short-term prediction. During 3 months following the 1^st^, 2^nd^ and 3^rd^ assessments, subjects who exhibited SB as a whole and SB with SI were 17 (25%) and 10 (11%), 16 (20%) and 7 (9%), and 21 (21%) and 6 (8%), respectively. SB as a whole during the subsequent 3 months was associated with SF-8 PCS score in the 1^st^ and 3^rd^ assessments (Exp (B) 0.884, 95% CI 0.828 - 0.944, p<0.001, and Exp (E) 0.957, 95% CI 0.917 - 0.999, p=0.046, respectively). SB with SI during the subsequent 3 months was associated with SF-8 PCS score in the 1^st^ assessment (Exp (B) 0.865, 95% CI 0.795 - 0.942, p=0.001). No significant association was found between any SB and other variables of follow-up assessments. The results indicated, despite some inconsistency, that SF-8 PCS score could have predictive value for SB as a whole and SB with SI during the subsequent 3-month period.

## Discussion

### Post-hospitalization course and SB incidence

This study recognized a very high incidence of SB as a whole, suicide attempt (SB with SI) and suicide for the total cohort within the 2-year post-hospitalization period of psychiatric suicidal patients: 67%, 38% and 6%, respectively. In contrast to previous studies, this study did not find a decline in SB or suicide incidence after a lapse of 1 year after discharge [4,6,8]. Results also indicated that mental health condition and hopelessness assessed by SF-8 MCS and BHS 4-item version scales improved during the period. However, the improvement seemingly did not prevent SB recurrence.

The SB recurrence rate in this study was higher than most of those reported in previous studies. A review by Owens et al. [9] indicated that the SB recurrence rate for patients who appeared in medical settings with SB within 1 year was around 16%. Within a longer time span of 8 years, Haukka et al. [6] calculated a suicide attempt incidence of 30% by using a nationwide study sample. In contrast, Links et al. [16] reported an exceptionally high SB recurrence rate of 39% within a 6-month after discharge period of psychiatric patients with suicidal ideation or a history of suicide attempt. One possible reason for the high recurrence rates in the study of Links et al. and this study would be that the large majority of patients in the samples exhibited chronic suicidality and presented more than 1 psychiatric disorder that required hospitalization.

### Predictive signs of SB

The present study selected anxiety disorders and cluster B PD as possible predictors for recurrence of SB as a whole, and cluster A PD for suicide attempt (SB with SI). Several previous studies have indicated that the disorders are possible predictive factors for SB. Reports of Sareen et al. [45] and Bolton et al. [46] from a large population-based prospective study demonstrated that anxiety disorders, and borderline PD (a type of cluster B), paranoid PD (a type of cluster A) and other PDs were antecedent of SB incidence. Borderline and antisocial PDs (types of cluster B) have been commonly recognized as a major underlying factor for SB [47]. Follow-up studies of Brent et al. [22] and Greenfield et al. [21] also stressed the importance of borderline PD. Additionally, other PD types have been highlighted as a risk factor. A prospective developmental cohort study of Johnson et al. [48] demonstrated that adolescent PDs, especially cluster C PDs, were predictive of suicide attempt in adulthood. Further studies are needed to draw a clearer conclusion as to which types of psychiatric disorders are relevant for predicting SB in specified populations.

A number of previous studies have stressed that depressive disorders or symptoms play a major role in SB repetition [22-24]. Suominen et al. [49] reported a high SB recurrence rate of 31% within a 4-year follow-up period of suicidal patients with depressive disorders. In the present study, severity of depressive symptoms and hopelessness were found to be significant factors for suicide attempt, as previous studies reported [16,20,27,29]. However, intensity of SI at the time of index SB episode did not have a significant association with SB recurrence in this study, whereas some previous studies indicated its positive association with subsequent SB [16,23].

Socio-demographic and life-historical characteristics of a history of previous SB, younger age, maltreatment in the developmental period and unemployment were indicated in this study as possible predictors of SB as a whole. Moreover, the stepwise multiple regression analysis selected life-historical and demographic factors: number of lifetime SBs, maltreatment in the development and younger age as more essential in SB prediction than psychiatric symptoms and diagnoses. Numerous studies confirmed the significance of a history of SB as a predictor of ensuing SB [6,21,22,24]. The association of SB repetition with childhood and adolescent maltreatment found in this study is consistent with findings of many studies indicating that maltreatment often anteceded SB [50]. The study of Johnson et al. [51] confirmed a link between maltreatment and SBs. Likewise, follow-up studies of Söderberg et al. [31] and Brent et al. [22] showed frequent SB recurrence of suicidal patients who had experienced sexual maltreatment during the developmental period. Regarding younger age, many studies showed that SB was common among youths [52]. Follow-up studies that examined SB of depressive patients have indicated that SB repetition is a characteristic of young individuals [22,23]. The influence of younger age on SB repetition may be understood as a result of youth-related impulsivity, which a developmental cohort study of Kasen et al. [53] demonstrated. Unemployment has also been referred to as a risk factor of SB in many studies [18,19,25]. A developmental cohort study of Fergusson et al. [54] showed that unemployment had an influence on subsequent SB. Regarding predictive signs of suicide attempt, however, depressive symptoms appeared to be predominant over socio-demographic characteristics, life-historical factors and psychiatric disorders.

This study revealed that SB as a whole and suicidal attempt (SB with SI) showed rather different patterns of predictive signs. It could be interpreted that life-historical and life-situational factors have an influence on SB as a whole while depressive symptoms, especially hopelessness intensify SI that accompanied SB. Further studies need to explore the difference in clinical meanings of the types of SB.

Additionally, this study indicated that poor physical health assessed by SF-8 health survey was a possible short-term predictive sign of SB recurrence. Some studies [55-57] demonstrated that physical symptoms were related to suicidality independent of depression, although they might be seen as a part of depressive symptomatology. Moreover, a follow-up study of discharged suicidal patients by Colman et al. [24] indicated a predictive value of physical symptoms. Therefore, there is a possibility that physical symptoms could be a critical target for treatment and suicide prevention for patients with SB. Additionally, the finding of this study that SF-8 MCS score and BHS 4 item version score did not have value in short-term SB prediction may explain that their improvement recognized in this study seemingly did not have an effect of SB reduction.

### Suicide incidence and characteristics of suicide victims

The suicide rate of this study of 6% within a 2-year period was higher than those of previous follow-up studies of suicidal patients. According to Owens et al. [9], suicide rates for patients who appeared with SB in emergency or medical settings were 2% within 1 year and 7% within 9 years. However, some previous studies indicated much higher suicide rate of approx. 3% in at high risk patients within a year after psychiatric hospitalization [5], which are comparable to the figure of this study. Links et al. [16] reported 3.3% within 6 months for psychiatric patients with suicidal ideation or a history of attempted suicide. Quite high suicide risks found in these studies could be explained by a multiplicity of risk factors, such as severe psychopathology and suicidality of the studied samples.

Schizophrenia and mood disorders have been noted as psychiatric disorders closely related to high suicide risk after hospitalization [6-8]. In this study, suicide rates within 2 years of patients with psychotic disorders and mood disorders were 12% (3/26) and 4.3% (3/70), respectively. Previous follow-up studies conducted on suicidal patients with specific diagnoses reported high suicide rates comparable to those of this study. Tidemalm et al. [7] reported that 18% of patients with schizophrenia and 14% of patients with mood disorders completed suicide within 1 year after discharge from hospitalization due to suicide attempt. In the study of Walsh et al. [58], a suicide rate of approx. 10% during a 2-year post-hospitalization period was calculated for patients with schizophrenia and a history of a recent suicide attempt. Regarding suicidal patients with depression, Suominen et al. [49] reported a high suicide rate of 6% in a 4-year follow-up period.

In addition, anxiety disorders and PDs should be considered as relevant disorders linked to impending SB or suicide. In the study of Tidemalm et al. [7], suicidal patients with anxiety disorders and those with PDs constituted the 2nd highest risk group following those with schizophrenia and with mood disorders. Other studies also indicated an increased risk of suicide in the affected patients [1,47]. In the present study, 3 female suicide victims with mood disorders had comorbid anxiety disorders and cluster B PDs. Previous research also indicated that among patients with depressive disorder, the comorbidity of anxiety disorders or borderline PD may increase the risk of SB [25,59,60]. The importance of this type of comorbidity warrants attention in future suicide prevention activities.

### Strengths and limitations

Strengths and limitations of the present study are as follows. Firstly, the strength resides primarily in its prospective study design, which could reduce recall biases that might have affected the results. The next is the full application of SCID I and II at entry, which provided a basis to deal with particular psychiatric diagnoses of the subjects, whereas few previous studies used structured diagnostic interview procedures. Additionally, it is to be noted that a permissible retention rate during the follow-up was attained by conducting serial assessments at a fixed interval.

The major limitation of the present study is a small sample size. When interpreting the findings, type II errors should be taken into account. Next, the studied sample may not be representative of suicidal patients admitted to a psychiatric hospital. In particular, a difference was found in the rates of psychotic disorders between the studied patients and those who did not participate. Restriction of the subjects’ residence to an area in Tokyo might also compromise its generalizability. Third, some measures used in the follow-up assessments might be too simplistic. Future studies should apply well-validated measures, particularly for social support, quality of treatment and distinguishing various SB types.

## Conclusions

The present study elucidated that a very high SB and suicide risk persisted during the post-hospitalization period of psychiatric suicidal patients though improvement of some psychiatric symptoms was observed. It also identified patterns of predictive signs of SB as a whole and suicide attempt, which suggested that SB-predictive signs varied depending on types of SBs. The results indicated that SB as a whole was more strongly predicted by life-historical factors such as number of lifetime SBs and maltreatment in the developmental period rather than other factors such as relevant psychiatric disorders and socio-demographic factors. These findings justifiably lent support to some earlier processes in the suicide development theories. From this viewpoint, the importance of clinical efforts to prevent SB as a whole should be emphasized to stop the processes leading to suicide. In contrast, depressive symptoms at entry were predictive of suicide attempts during the follow-up period, which suggested that to mitigate the symptoms promptly might be contributory of preventing later intensification of SI. Additionally, poor physical health was presented as a novel candidate of proximal SB predictors for the patients. The predictive signs and characteristics are to be applied for further deepening understandings of SBs and developing individualized suicide prevention programs for the patients. It should also be stressed that clinical and research efforts be directed to improving treatments and to examining their effectiveness for this patient population at extremely high risk of suicide.

## Abbreviations

SB: suicidal behavior; SI: suicidal intent; DSM-IV: Diagnostic and Statistical Manual of Mental Disorders, Fourth Edition; SF-8 health survey: a generally used abbreviated name of “8 item Short Form health survey”; TMMH: Tokyo Metropolitan Matsuzawa Hospital; TMCMW: Tokyo Metropolitan Chubu Center for Mental Health and Welfare; SCID-I: CV, Structured Clinical Interview for DSM-IV Axis I Disorders, Clinician Version; SCID-II: Structured Clinical Interview for DSM-IV Axis II Personality Disorders; SIS: Suicide Intent Scale; BDI: Beck Depression Inventory-II; BHS: Beck Hopelessness Scale; SF-8 PCS score: SF-8 Physical Component Summary score; SF-8 MCS score: SF-8 Mental Component Summary score.

## Competing interests

The authors declare that they have no competing interests.

## Authors' contributions

NH conceptualized and designed the study, collected the data, performed the statistical analysis, and drafted the manuscript. MI, AI, YY, KU, YI, TT and KI conceptualized and designed the study, collected the data. HH, YT, NK, MN and YY conceptualized and designed the study. HI performed statistical analysis. All authors read and approved the final manuscript.

## Pre-publication history

The pre-publication history for this paper can be accessed here:

http://www.biomedcentral.com/1471-244X/12/186/prepub
